# Ethanol Extract of* Cudrania tricuspidata* Leaf Ameliorates Hyperuricemia in Mice via Inhibition of Hepatic and Serum Xanthine Oxidase Activity

**DOI:** 10.1155/2018/8037925

**Published:** 2018-12-02

**Authors:** Seung-Hui Song, Dae-Hun Park, Min-Suk Bae, Chul-Yung Choi, Jung-Hyun Shim, Goo Yoon, Young-Chang Cho, Deuk-Sil Oh, In-Soo Yoon, Seung-Sik Cho

**Affiliations:** ^1^Department of Pharmacy, College of Pharmacy, Mokpo National University, Muan, Jeonnam 58554, Republic of Korea; ^2^Department of Nursing, Dongshin University, Naju, Jeonnam 58245, Republic of Korea; ^3^Department of Environmental Engineering, Mokpo National University, Muan, Jeonnam 58554, Republic of Korea; ^4^Department of Natural Medicine Research, Jeonnam Institute of Natural Resources Research, Jangheung, Jeonnam 59338, Republic of Korea; ^5^College of Pharmacy, Chonnam National University, Buk-gu, Gwangju 61186, Republic of Korea; ^6^Jeonnam Forest Resource Research Institute, Naju, Jeonnam 58213, Republic of Korea; ^7^Department of Manufacturing Pharmacy, College of Pharmacy, Pusan National University, Geumjeong, Busan 46241, Republic of Korea

## Abstract

*Cudrania tricuspidata* Bureau (Moraceae) (CT) is a dietary and medicinal plant distributed widely in Northeast Asia. There have been no studies on the effect of CT and/or its active constituents on* in vivo* xanthine oxidase (XO) activity, hyperuricemia, and gout. The aim of this study was to investigate XO inhibitory and antihyperuricemic effects of the ethanol extract of CT leaf (CTLE) and its active constituents* in vitro* and* in vivo*. Gas chromatography-mass spectrometry (GC-MS) and high-performance liquid chromatography (HPLC) analyses were used to determine a chemical profile of CTLE. XO inhibitory and antihyperuricemic effects of CTLE given orally (30 and 100 mg/kg per day for 1 week) were examined in potassium oxonate-induced hyperuricemic ICR mice. CTLE exhibited XO inhibitory activity* in vitro* with an IC_50_ of 368.2 *μ*g/mL, significantly reduced serum uric acid levels by approximately 2-fold (7.9 nM in normal mice; 3.8 nM in 30 mg/kg CTLE; 3.9 nM in 100 mg/kg CTLE), and significantly alleviated hyperuricemia by reducing hepatic (by 39.1 and 41.8% in 30 and 100 mg/kg, respectively) and serum XO activity (by 30.7 and 50.1% in 30 and 100 mg/kg, respectively) in hyperuricemic mice. Moreover, several XO inhibitory and/or antihyperuricemic phytochemicals, such as stigmasterol, *β*-sitosterol, vitamin E, rutin, and kaempferol, were identified from CTLE. Compared with rutin, kaempferol showed markedly higher XO inhibitory activity* in vitro*. Our present results demonstrate that CTLE may offer a promising alternative to allopurinol for the treatment of hyperuricemia and gout.

## 1. Introduction

Hyperuricemia is defined as abnormally high levels of uric acid in the blood stream. In a chronic hyperuricemic state, uric acid can be crystallized and deposited as monosodium urate in the joints. This can cause inflammatory arthritis with severe pain, which is defined as gout [1]. Thus, hyperuricemia has been regarded as the main etiological factor in gout and the first phases in the process of gout [2]. Worldwide, the prevalence of gout is reported to be approximately 0.1%-10%, although it is increasing in both developed and developing countries [3]: more than two million people in the United States are reportedly afflicted with gout [4] and the prevalence of gout and hyperuricemia in China was 1.1% and 13.3%, respectively [5]. Moreover, patients with gout or hyperuricemia have a much higher risk for the development of various comorbidities, such as hypertension, metabolic syndromes, and cardiovascular diseases [6–9].

As xanthine oxidase (XO) is a key enzyme involved in the purine nucleotide catabolism from hypoxanthine to uric acid, both uric acid and XO have been regarded as the main biochemical index and relevant therapeutic target for hyperuricemia and gout [10]. Thus, the management of hyperuricemia and gout is primarily aimed at the modulation of the activity of XO. Allopurinol is a representative XO inhibitor, used primarily for the treatment of hyperuricemia and gout. However, allopurinol induces hypersensitivity reactions with a probability of up to 2%, some of which can be severe, with a mortality of up to 20% [11]. Moreover, other adverse reactions associated with allopurinol include fetal liver necrosis, eosinophilia, Stevens-Johnson syndrome, and nephropathy [12]. Owing to these unmet medical needs, the development of alternative herbal medicines for the treatment of hyperuricemia and gout is an active field of study [13].

*Cudrania tricuspidata* Bureau (Moraceae) (CT) is a dietary and medicinal plant which is distributed widely in Northeast Asia (South Korea, Japan, and China) [14]. The Donguibogam, an essential bible of Asian traditional medicine, reported that the daily intake of CT stem or leaf extracts at doses of 50–70 g can ameliorate inflammation and pain. Several previous studies reported that the root, fruit, and/or leaf of CT possessed various pharmacological activities, such as anti-inflammatory, cytotoxic, antiobesity, and hepatoprotective activity [15–19]. We previously prepared an ethanolic extract of CT leaf and optimized its extraction conditions with respect to various biological activities [15]. In the previous study, the optimized CT leaf extract (CTLE) was found to possess* in vitro* XO inhibitory activity [15]. However, to the best of our knowledge, there have been no studies on the effect of CT and/or its active constituents on* in vivo* XO activity, hyperuricemia, or gout; hence, further investigation is required.

Therefore, this study aimed to investigate the XO inhibitory and antihyperuricemic effects of CTLE and its constituent bioactive phytochemicals. Gas chromatography-mass spectrometry (GC-MS) and high-performance liquid chromatography (HPLC) analyses were used to determine chemical profile. XO inhibitory and antihyperuricemic effects were evaluated by using a relevant* in vivo* mouse model of hyperuricemia and the* in vitro* enzymatic system.

## 2. Materials and Methods

### 2.1. Plant Materials

CT leaves were collected in Jeonnam Forest Resource Institute (Naju, South Korea) in May 2017 and identified by Dr. Deuk-Sil Oh affiliated to the Jeonnam Forest Resource Institute. A voucher specimen (MNUCSS-CT-01) was stored in the Mokpo National University (Muan, South Korea). The leaves were processed as previously described [15]. Briefly, the air-dried, powdered CT leaves (100 g) were extracted twice with 1 L ethanol at approximately 20°C (room temperature) for 72 h. The resultant ethanolic solution was filtered, evaporated, and freeze-dried to give CTLE.

### 2.2. Animals

Four-week-old male ICR mice were obtained from Orient Bio, Co. (Sungnam, South Korea). They were bred at 20-24°C (room temperature), with 12 h light (07:00-19:00) and dark (19:00-07:00) cycles, and a relative humidity of 50% ± 5% in a clean rodents' facility. The mice were housed in ventilated mice cages (Tecniplast USA, Inc.) with filtered and pathogen-free air. Water and laboratory mouse pellet food (Agribrands Purina Korea, Inc.) were provided* ad libitum*. All animal procedures were approved by the Institutional Animal Care and Use Committee (IACUC) of Jeonnam Bioindustry Foundation (approval number: JINR1503) and conducted in full compliance with the IACUC guidelines.

### 2.3. Chemical Profiling by GC-MS and HPLC Analyses

GC-MS analysis was performed as reported previously with slight modifications [20]. An Agilent 7890 gas chromatograph system was coupled to a quadrupole Agilent 5975C electron ionization (70 eV) mass spectrometric detector (Agilent Technologies, Palo Alto, CA, USA). The operational parameters for the GC-MS analysis are listed in Table 1. In addition, we purified and identified two constituents (rutin and kaempferol) by using preparative column liquid chromatography (prep-LC) and preparative thin layer chromatography. The analytical conditions for prep-LC are summarized in Table 1. Then, constituent profiling of CTLE was performed by using an Alliance 2695 HPLC system (Waters; Milford, MA, USA) coupled to a photodiode array detector at the wave length of 320 nm. An Agilent Zorbax extended C18 analytical column (150 mm × 5 mm; 5 *μ*m) was used with a filtered and degassed mobile phase that consisted of solvents A (acetonitrile) and B (0.2% phosphoric acid). Gradient elution (from 10/90 to 100/0, v/v) was performed at flow rate of 0.8 mL/min. The column temperature and sample injection volume were 25°C and 10 *μ*L, respectively.

### 2.4. Determination of In Vitro Xanthine Oxidase (XO) Inhibitory Activity

XO inhibitory activity was determined through the measurement of uric acid formation in the XO enzyme assay system, as described previously [21]. The enzyme reaction mixture (total volume: 1000 *μ*L) consisted of 100 *μ*L XO (0.2 U/mL), 200 *μ*L xanthine (1 mM dissolved in 0.1 N NaOH), 100 *μ*L analysis sample or allopurinol (positive control), and 600 *μ*L phosphate buffer (100 mM; pH 7.4). The enzyme reaction was initiated through the addition of the enzyme and terminated through the addition of an aliquot of 1 N HCl (0.2 mL). The changes in the absorbance of the reaction mixture in comparison with the absorbance of blank were monitored at 290 nm for 15 min by using a UV/Vis spectrophotometer (PerkinElmer, Inc., Waltham, MA, USA). The IC_50_ of CTLE for the inhibition of* in vitro* XO activity was determined by nonlinear regression using GraphPad Prism 5.01 (GraphPad Software, San Diego, CA) according to the following equation:

(1) $$\begin{array}{c} Y=min+\frac{\max-\min}{1+{\left(X/{IC}_{50}\right)}^{-P}} \end{array}$$

where X and Y are the inhibitor (CTLE) concentration and response, respectively. Max and Min are the initial and final Y value, respectively, and the exponent P represents the Hill coefficient.

### 2.5. Pretreatment and Hyperuricemia Induction in Mice

CTLE or ALP was suspended in 0.3% carboxymethylcellulose (CMC) aqueous solution. The mice were divided into five groups (*n* = 5 for each group) and pretreated orally once per day for 7 days, as follows: the mice in two negative control groups received 0.3% CMC aqueous solution (NOR and HU groups); the mice in the positive control group received ALP suspension at 10 mg/kg (ALP group); the mice in CTLE30 and CTLE100 groups received CTLE suspension at 30 and 100 mg/kg, respectively. To induce hyperuricemia, all mice, except those in the NOR group, were intraperitoneally given potassium oxonate (uricase inhibitor; 250 mg/kg dissolved in PBS) 1 h prior to the final pretreatment on day 7 (mice in the NOR group received PBS instead of potassium oxonate) [22]. Finally, 1 h after the final pretreatment on day 7, approximately 500 *μ*L blood was collected via the tail vein, allowed to clot at 4°C for 1 h, and centrifuged at 10,000 *g* for 15 min to obtain serum. The resultant serum samples were stored at -80°C for further analysis.

### 2.6. Determination of In Vivo Serum Uric Acid Concentration and XO Activity

Serum concentration of uric acid was determined by using a standard diagnostic kit (Abcam; Cambridge, UK). The activity of XO in mouse liver and serum (expressed as micromoles of uric acid formed per minute (U) per milligram protein) was spectrophotometrically determined through the measurement of formation of uric acid from xanthine (in triplicate) [23]. Briefly, mouse liver (0.5 g) was homogenized in 1 mL sodium phosphate buffer (50 mM; pH 7.4). The homogenate was centrifuged at 3,000 *g* for 10 min at 4°C. After removal of the lipid layer, the supernatant was centrifuged at 10,000 *g* for 60 min at 4°C. The resultant supernatant was used as the analysis sample for the analysis of XO residual activity and total protein concentration. To measure XO activity, 10 *μ*L of sample was transferred into a test tube containing 540 *μ*L of 1 mM potassium oxonate solution dissolved in sodium phosphate buffer (50 mM; pH 7.4) and incubated at 35°C for 15 min. Then, the reaction was initiated through the addition of 120 *μ*L of xanthine solution (250 mM). After 0 and 30 min, the reaction was stopped through the addition of 100 *μ*L of HCl (600 mM), and the test tube was centrifuged at 3,000 *g* for 5 min. The absorbance of the supernatant was measured at 295 nm by using the UV/Vis spectrophotometer. The total protein concentration of the sample was measured by using the Bradford method [20].

### 2.7. Statistical Analysis

To analyze the differences between two means of unpaired data, Student's* t*-test was performed. To analyze the differences among three or more means of unpaired data, analysis of variance (*post hoc* test: Tukey's HSD test) was performed. A* p*-value less than 0.05 was considered to be statistically significant. All data were expressed as the mean ± standard deviation and rounded to three significant figures.

## 3. Results

### 3.1. In Vitro XO Inhibitory Effect of CTLE

**Figure 1:**
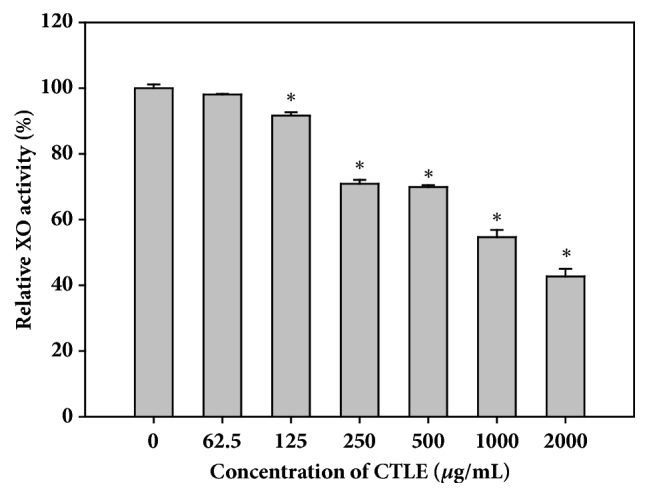
*In vitro* xanthine oxidase (XO) inhibitory activity of CTLE at concentrations between 0 and 2000 *μ*g/mL. The rectangular bars and their error bars represent the means and standard deviations, respectively (*n* = 5). The asterisks indicate values that are significantly different from those of the control (0 *μ*g/mL) group (*p* < 0.05).

Figure 1 shows the XO inhibitory effect of CTLE at various concentrations. CTLE at 125 *μ*g/mL or higher significantly inhibited the* in vitro* XO activity, whereas CTLE at 62.5 *μ*g/mL did not. CTLE inhibited XO activity in a concentration-dependent manner. The IC_50_ of CTLE for the inhibition of XO activity was estimated to be 368.2 *μ*g/mL.

### 3.2. Effect of CTLE on In Vivo Serum Uric Acid Levels

**Figure 2:**
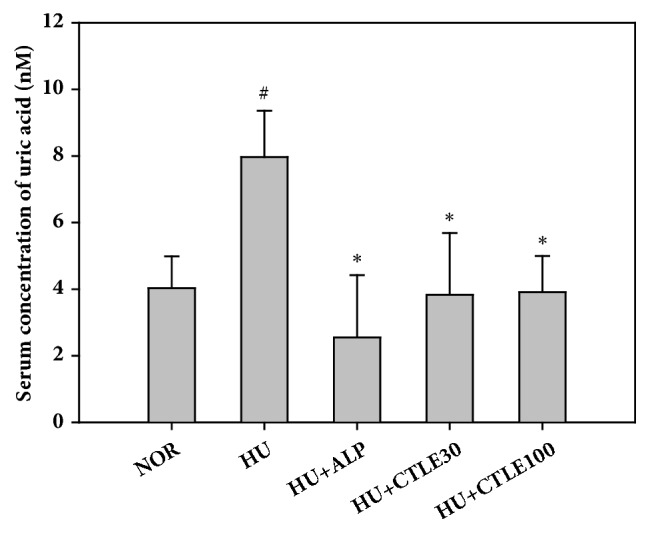
Serum uric acid levels after the oral administration of saline in normal mice (NOR) and after the oral administration of saline (HU), allopurinol at a dose of 10 mg/kg (HU+ALP), or CTLE at doses of 30 mg/kg (HU+CTLE30) and 100 mg/kg (HU+CTLE100) for 7 days prior to the induction of hyperuricemia in mice. The rectangular bars and their error bars represent the means and standard deviations, respectively (*n* = 5). The asterisks and pound sign indicate values that are significantly different from those of the HU and NOR group, respectively (*p* < 0.05).

Figure 2 shows the effect of CTLE on the serum concentrations of uric acid in hyperuricemic mice. At 1 h after intraperitoneal injection of potassium oxonate, the serum uric acid concentrations in the hyperuricemic control mice group were significantly higher than those in the normal control mice group, by approximately 2-fold (7.9 ± 1.4 nM* versus* 4.0 ± 1.0 nM), which indicated that the mouse model of hyperuricemia was successfully established, as reported previously [22]. The serum uric acid concentrations in the normal control mice group were comparable to those in the hyperuricemic mice groups that were pretreated with allopurinol or CTLE at doses of 30 and 100 mg/kg for 1 week prior to the induction of hyperuricemia. The serum uric acid levels in allopurinol-pretreated (HU+ALP) and CTLE-pretreated hyperuricemic mice groups (HU+CTLE30 and HU+CTLE100) were 2.5 ± 1.9 nM, 3.8 ± 1.9 nM, and 3.9 ± 1.1 nM, respectively.

### 3.3. Effect of CTLE on In Vivo Hepatic and Serum XO Activity

**Figure 3:**
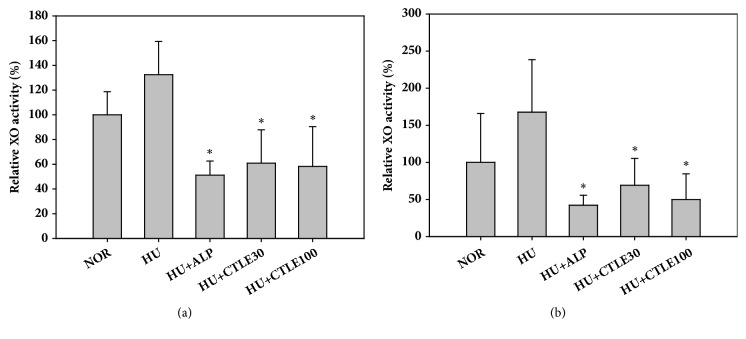
Relative activity of hepatic (a) and serum (b) xanthine oxidase (XO) after the oral administration of saline in normal mice (NOR) and after the oral administration of saline (HU), allopurinol at a dose of 10 mg/kg (HU+ALP), or CTLE at doses of 30 mg/kg (HU+CTLE30) and 100 mg/kg (HU+CTLE100) in hyperuricemic mice for 7 days. The rectangular bars and their error bars represent the means and standard deviations, respectively (*n* = 5). The asterisks indicate values that are significantly different from those of the HU group (*p* < 0.05).

The effects of CTLE on hepatic and serum XO activity in hyperuricemic mice are shown in Figure 3. There was no significant difference in hepatic or serum XO activity between the normal and hyperuricemic control mice groups. However, hepatic XO activity in hyperuricemic mice was significantly reduced by the 1-week oral pretreatment of allopurinol (by 48.8%) or 30 and 100 mg/kg CTLE (by 39.1 and 41.8%, respectively), as shown in Figure 3(a). Similarly, serum XO activity in hyperuricemic mice was significantly reduced by the 1-week oral pretreatment of allopurinol (by 57.7%) or 30 and 100 mg/kg CTLE (by 30.7 and 50.1%, respectively), as shown in Figure 3(b).

### 3.4. Chemical Profiling by GC-MS and HPLC Analyses

**Figure 4:**
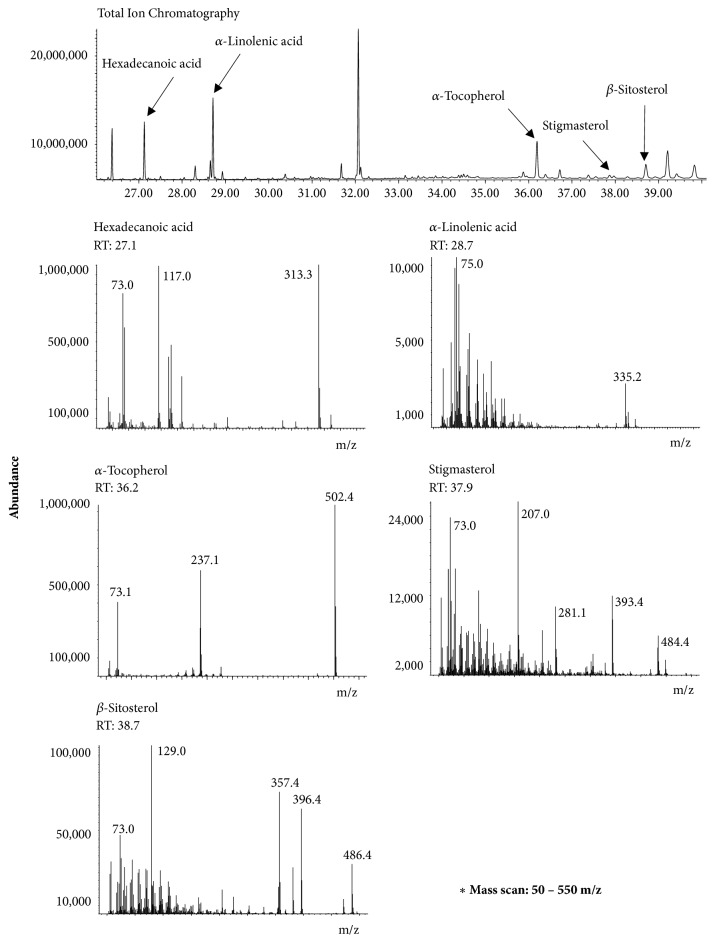
Representative GC-MS chromatogram of CTLE.

**Figure 5:**
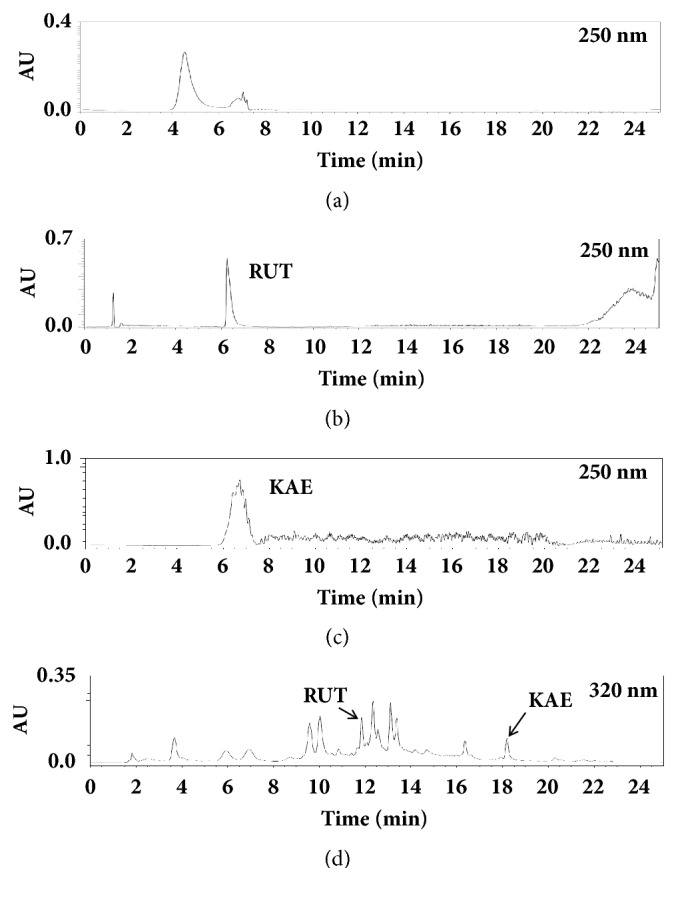
Representative chromatographic profiles showing the bioactive constituents of CTLE. (a) Elution profile of CTLE using prep-LC; (b) elution profile of purified rutin (RUT) using prep-LC; (c) elution profile of purified kaempferol (KAE) using prep-LC; (d) HPLC chromatogram of CTLE.

GC-MS and HPLC analyses were performed to identify bioactive phytochemicals from CTLE. Representative GC-MS and HPLC chromatograms of phytochemicals with their retention times are shown in Figures 4 and 5, respectively. The contents of the phytochemicals identified by GC-MS and HPLC analyses are listed in Table 2. Linolenic acid, vitamin E, hexadecanoic acid, *β*-sitosterol, and stigmasterol were identified by GC-MS analysis, whereas rutin and kaempferol were identified by HPLC analysis.

### 3.5. In Vitro XO Inhibitory Effects of Phytochemicals Identified by HPLC Analysis

**Figure 6:**
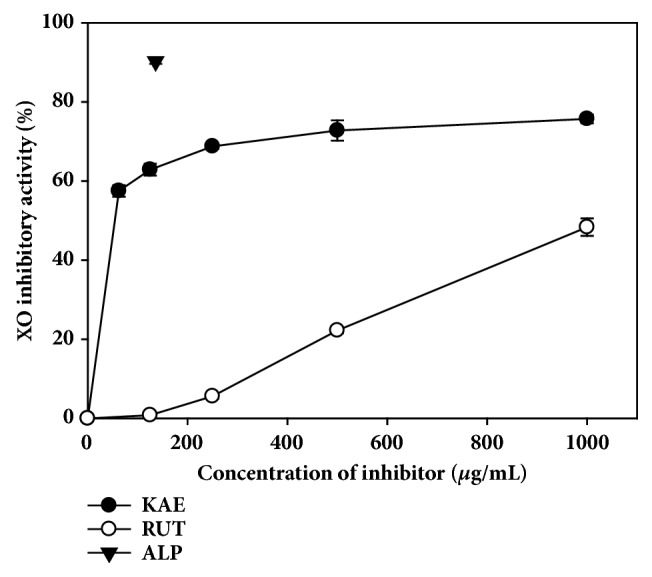
Xanthine oxidase (XO) inhibitory activity of allopurinol (ALP) and bioactive phytochemicals identified from CTLE (kaempferol, KAE; rutin, RUT). The bullet symbols and their error bars represent the means and standard deviations, respectively (*n* = 5). ALP was tested at a concentration of 136 *μ*g/mL, and KAE and RUT were tested at concentrations between 0 and 1000 *μ*g/mL.

The concentration dependent* in vitro* XO inhibitory activity of the two flavonoids identified by HPLC analysis is shown in Figure 6. Compared with rutin, kaempferol showed notably higher levels of XO inhibitory activity within the concentration range of the compounds tested.

## 4. Discussion

This study has provided novel experimental data on the antihyperuricemic effects of CTLE and its constituent active phytochemicals. In our previous study, the conditions for the ethanolic extraction of CT leaf were evaluated to provide optimum biological activity and chemical profile [15]. As a result, CT leaf extract prepared with 100% ethanol exhibited the highest total flavonoid content and XO inhibitory activity [15]. XO inhibitory activity in several plant extracts has been attributed to the presence of flavonoids [24]. Thus, the 100% ethanolic extract (CTLE) was selected and further evaluated for antihyperuricemic potential in the present study.

As shown in Figures 2 and 3, CTLE at doses of 30 and 100 mg/kg significantly reduced serum uric acid levels and inhibited hepatic and serum XO activities in hyperuricemic mice. These results clearly showed that the 1-week pretreatment with oral CTLE significantly alleviated the hyperuricemic state in mice. Meanwhile, the average values of hepatic and serum XO activity were slightly higher in the hyperuricemic control mice group than in the normal mice group, but the differences were not statistically significant (*p* = 0.38 for hepatic XO activity and 0.33 for serum XO activity). This suggested that the intraperitoneal administration of potassium oxonate, an uricase inhibitor, did not significantly modulate the XO activity in mice.

As shown in Table 2, several bioactive constituents related to XO-inhibitory, antihyperuricemic, and/or anti-gout properties were identified from CTLE by GC-MS and HPLC analyses. Ferraz-Filha et al. (2016) reported that stigmasterol reduced serum uric acid levels through the inhibition of hepatic XO activity in hyperuricemic mice. It was also reported that stigmasterol and *β*-sitosterol alleviated the local paw edema induced by monosodium urate crystals in mice [25, 26]. Mohd Fahami et al. (2012) reported that vitamin E, a well-known antioxidant, exhibited gastroprotective effects through the reduction in gastric XO activity in rats. There have been no previous studies related to the effects of linolenic acid and hexadecanoic acid on XO activity, hyperuricemia, and/or gout. However, it is well known that these two phytochemicals have anti-inflammatory activity and are able to potentially modulate secondary inflammatory damage in gouty arthritis, which warrant further investigation [27, 28]. Additionally, we identified two different XO inhibitory flavonoids,* i.e.,* kaempferol and rutin, from CTLE. Kaempferol is a major flavonoid present widely in the diet and Chinese herbal medicines; it comprises 22%-29% of the total flavonoid intake [29]. Our present results showed that kaempferol could serve as a potential XO inhibitor, which was consistent with previous reports [30, 31]. Moreover, kaempferol was demonstrated to inhibit XO activity in a competitive manner through the insertion at the hydrophobic active site of XO to interrupt the entrance of substrate [30]. Previous studies also reported that rutin reduced serum uric acid levels, serum XO activity, and hepatic XO activity in hyperuricemic mice [32, 33]. However, compared with kaempferol, rutin exerted relatively lower* in vitro* XO inhibitory activity (Figure 6).

The effective dose levels found in this study (30 and 100 mg/kg) appear to be relatively lower than those reported in previous studies on plant extracts with antihyperuricemic activity (100-978 mg/kg) [34–37]. The daily mouse doses of 30 and 100 mg/kg can be converted into human equivalent doses of 146 and 487 mg/60 kg human/day, based on a conversion factor of 12.33 [38]. As the process yield for the preparation of CTLE was 16.9%, approximately 1-3 g of raw plant materials would be required for the preparation of each CTLE dose in a clinical study. These are feasible and advantageous circumstances for the industrial development of dietary or medicinal formulations containing CTLE.

## 5. Conclusions

This study demonstrated that relatively low dose of CTLE (30 and 100 mg/kg) significantly alleviated hyperuricemia through the reduction of serum and hepatic XO activity. Moreover, CTLE was found to contain several XO inhibitory and/or antihyperuricemic phytochemicals such as stigmasterol, *β*-sitosterol, vitamin E, rutin, and kaempferol. To the best of our knowledge, this is the first report on the XO inhibitory and antihyperuricemic effects of CTLE and its active phytochemicals. Our present results have shown that CTLE may offer a promising alternative to allopurinol for the treatment of hyperuricemia and gout.

## Figures and Tables

**Table 1:** Analytical conditions of the GC-MS and preparative HPLC methods.

**Parameter**	**Condition**			
	*GC-MS*			
Column	Agilent HP-5MS fused silica capillary column			
	(30 m × 0.25 mm *i.d.*, 0.25 *μ*m film thickness)			
Carrier	Helium			
Split	1 : 5			
Injection volume	1 *μ*L			
MS source	230°C			
MS quad	150°C			
Analytical temperature		Rate	Value	Hold time
	Initial		65°C	10 min
	Ramp	10°C/min	300°C	22 min
	Total	55.5 min		
Thermal aux	300°C			
Electron ionization	70 ev			
Mass range	50–550 amu			
Scan method	Full scan			

	*Preparative HPLC*			
Column	OP C18-51002510 (250×10.0 mm, 5 *μ*m)			
Flow rate	3 mL/min			
Injection volumn	1000 *μ*L			
UV detection	250 nm			
Run time	25 min			
Gradient flow	Time (min)	ACN (v/v%)	0.2% phosphoric acid (v/v%)	
	0	10	90	
	5	10	90	
	18	50	50	
	20	100	0	
	21	10	90	
	25	10	90	

**Table 2:** The main phytochemicals identified from CTLE.

Constituent	Content (%)
*GC-MS*	
Linolenic acid	6.92
Vitamin E	5.62
Hexadecanoic acid	4.56
*β*-sitosterol	2.93
Stigmasterol	0.6
*HPLC*	
Rutin	0.44
Kaempferol	0.27

## Data Availability

No data were used to support this study.
